# Familial and sporadic idiopathic pulmonary fibrosis: making the diagnosis from peripheral blood

**DOI:** 10.1186/1471-2164-15-902

**Published:** 2014-10-16

**Authors:** Eric B Meltzer, William T Barry, Ivana V Yang, Kevin K Brown, Marvin I Schwarz, Hamish Patel, Allison Ashley, Paul W Noble, David A Schwartz, Mark P Steele

**Affiliations:** Department of Medicine, Duke University Medical Center, Durham, NC USA; Department of Biostatistics and Computational Biology, Dana-Farber Cancer Institute, Boston, MA USA; Department of Medicine, Anshutz Medical Campus, University of Colorado, Aurora, CO USA; National Jewish Health, Denver, CO USA; Edward Via College of Osteopathic Medicine, Spartanburg, SC USA; Department of Medicine, Cedars-Sinai, Los Angeles, CA USA; Division of Allergy, Pulmonary, and Critical Care, Vanderbilt University Medical Center, 1313 21st Avenue South, 1105 Oxford House, Nashville, TN USA

**Keywords:** IPF, FIP, gene signature, Bayesian probit regression

## Abstract

**Background:**

Peripheral blood biomarkers might improve diagnostic accuracy for idiopathic pulmonary fibrosis (IPF).

**Results:**

Gene expression profiles were obtained from 89 patients with IPF and 26 normal controls. Samples were stratified according to severity of disease based on pulmonary function. The stratified dataset was split into subsets; two-thirds of the samples were selected to comprise the training set, while one-third was reserved for the validation set. Bayesian probit regression was used on the training set to develop a gene expression model for IPF versus normal. The gene expression model was tested by using it on the validation set to perform class prediction. Unsupervised clustering failed to discriminate between samples of different severity. Therefore, samples of all severities were included in the training and validation sets, in equal proportions. A gene signature model was developed from the training set. The model was built in an iterative fashion with the number of gene features selected to minimize the misclassification error in cross validation. The final model was based on the top 108 discriminating genes in the training set. The signature was successfully applied to the validation set, ROC area under the curve = 0.893, p < 0.0001. Using the optimal threshold (0.74) accurate class predictions were made for 77% of the test cases with sensitivity = 0.70, specificity = 1.00.

**Conclusions:**

By using Bayesian probit regression to develop a model, we show that it is entirely possible to make a diagnosis of IPF from the peripheral blood with gene signatures.

**Electronic supplementary material:**

The online version of this article (doi:10.1186/1471-2164-15-902) contains supplementary material, which is available to authorized users.

## Background

While IPF is a significant cause of morbidity and mortality worldwide, the standard approach to diagnosing IPF can be quite challenging [1–3]. It requires integration of clinical, pathological and radiological data. A multidisciplinary discussion among expert clinician, radiologist and pathologist is required for high diagnostic accuracy [4]. It can be difficult to replicate that type of diagnostic approach outside of the academic setting [5]. In addition, the recent IPF guidelines warn that, even among experts, the diagnostic confidence of IPF must be qualified. In other words, the diagnosis of IPF must carry a modifier: definitive, probable or merely possible [1]. Given these conditions, at least 10% of all cases of interstitial lung disease (ILD) remain unclassified [6–9].

Meanwhile, there are families impacted by familial interstitial pneumonia (FIP) in which some family members are affected and others are unaffected [10]. Unaffected family members are at risk for eventually developing the disease; but, other than genetic testing which has not been standardized, there are no means for predicting this outcome. When an unaffected family member develops respiratory symptoms, a radiographic test or surgical lung biopsy must be performed to secure the diagnosis similar to the diagnostic approach in IPF. A blood-based diagnostic test would be useful in this context, to avoid unnecessary radiation or surgery, and to screen at risk family members for FIP. Currently, no blood tests exist for this purpose.

We hypothesize that an accurate diagnosis of IPF can be obtained from the peripheral blood by leveraging the transcriptome with a *functional gene signature.*

Here, it is important to distinguish between *functional gene signatures* and simple *gene lists*. The concept is that a *functional gene signature* is akin to a multiplex biomarker, in that a *functional gene signature* can be used to query unknown samples for the purpose of class prediction. *Functional gene signatures* can be specifically designed to predict diagnosis or prognosis. There are several components to any *functional gene signature*: (1) a set of training samples; (2) a specified feature list (select genes), upon which the model is built; and (3) the actual expression values of the selected genes, values that are specific to the training set. The final product is a regression equation (with intercept and coefficients for each genetic feature).

By contrast, a *gene list* is nothing more than an inventory of differentially-expressed genes. *Gene lists* serve a different purpose. They are designed to generate novel hypotheses or identify novel molecular targets and pathways; but *gene lists* cannot be used to make class predictions.

Few *functional gene signatures* have been published in the field of interstitial lung disease. Selman *et al.* published a gene signature, based on whole lung tissue, which distinguished IPF from hypersensitivity pneumonitis [11]. We recently published a whole lung tissue-based gene signature that distinguished IPF from normal controls by using Bayesian probit regression (BPR) to develop the signature [12].

Although it can be difficult to diagnose idiopathic pulmonary fibrosis (IPF), molecular biomarkers hold the promise of making IPF diagnoses more accurate. Here, we present an independently validated, peripheral blood-based, BPR-derived diagnostic gene signature for IPF.

## Methods

### Study population

One hundred thirty subjects were recruited through the Interstitial Lung Disease and Familial Pulmonary Fibrosis Programs at National Jewish Health and Duke University. This cohort has been previously described [13]. In brief, all subjects met the modified criteria of the ATS/ERS/JRS/ALAT for the diagnosis of IPF. [1] In familial cases, only one subject per family was included in this cohort. All subjects had no current tobacco use, no current use of prednisone, azathioprine, or other immune-modulating drugs, and provided written IRB-approved informed consent.

One hundred twenty three samples passed strict tests of RNA quality assurance [13]. Clinical annotation was not available for 8 subjects due to discrepancies between the genomic and quality control databases. Therefore, only 115 samples were included in this analysis; these 115 samples are a subset of the complete dataset (raw and processed data) that is available through the Gene Expression Omnibus (http://www.ncbi.nlm.nih.gov/geo/; accession number GSE33566).

Samples were assigned to the training cohort or the validation cohort by a stratified systematic method. First, samples were stratified by pulmonary function and disease severity (see the next section). Then, for each group by severity, every third patient was assigned to the validation cohort.

### Pulmonary function measures and categories of disease severity

Forced vital capacity (FVC) and diffusion capacity for carbon monoxide (DLCO) measurements were obtained in accordance with standard guidelines [14, 15]. When both measurements (FVC and DLCO) were available, mild disease was defined as having both the percent predicted FVC ≥ 75% and percent predicted DLCO ≥ 65%. Severe disease was defined as both FVC ≤ 50% and DLCO ≤ 35%. All other combinations of pulmonary function measurements were classified as moderate disease. When some of the pulmonary function data was missing, either FVC or DLCO alone was used to classify the severity. In eight cases, no pulmonary function data was available and disease severity remained unknown.

### Sample processing and expression profiling

#### Peripheral blood collection and RNA isolation

The collection of samples was previously described [13]. Briefly, peripheral blood was collected in PAXgene RNA tubes (PreAnalytiX, 762165). RNA extraction and purification was performed manually utilizing the PAXgene Blood RNA kit (PreAnalytiX, 762164) according to the manufacturer’s protocol. Quantification of total RNA was measured via the Nanodrop ND-1000 spectrophotometer (NanoDrop Technologies, Wilmington, DE). RNA quality was assessed with a RNA 6000 NanoChip (Agilent, Palo Alto, CA) on the 2100 Bioanalyzer (Agilent, Palo Alto, CA) by ratio comparison of the 18 S and 28 S rRNA bands.

#### Microarrays

Generation of the expression profiles from peripheral blood samples was previously described [13]. In summary, Agilent Whole Human Genome Oligonucleotide Microarrays (Agilent, Palo Alto, CA), were used to determine gene expression levels in peripheral blood. Total RNA was used as a template for synthesis of cDNA utilizing the One Color Low Input Agilent Quick Amp Labeling Kit and the Spike-In Kit to provide positive controls. The Agilent one-color microarray based gene expression analysis was followed per manufacturer’s instructions, and passed Agilent’s recommended quality control tests. Samples were run in batches (batch 1 through batch 6).

### Statistical analysis

#### Data processing

Expression estimates were normalized with the Agi44x4 package in the R computing environment as previously described [13]. Both the raw and processed datasets are available through the Gene Expression Omnibus (GSE33566).

#### Unsupervised analyses

Global gene expression profiles were filtered for the top 90th percentile by coefficient of variation, and samples were evaluated by principal component analysis (PCA), and genes and samples by agglomerative hierarchical clustering using average linkage and Pearson correlation coefficients.

#### Bayesian probit regression

Predictive models of IPF versus normal were derived by an established method [12]. Briefly, top differentially expressed features were selected using Student t-statistics and expression values were summarized by the first two eigenvalues from a singular value decomposition of the training samples. Summarized expression values were applied to a Bayesian probit regression model with non-informative priors for the parameters pertaining to the linear model and variance term. A Monte Carlo Markov Chain (MCMC) was used to obtain the posterior distribution for the linear predictor and regularized probabilities for each decomposed data set. The model was evaluated (internal validation) by leave-one-out cross-validation (LOOCV), whereby feature selection was repeated for each sample and the expected predicted probability was taken as the average value from the posterior distribution derived from the MCMC. External validation of performance was assessed using receiver operator characteristic (ROC) analyses and the Youden index to identify optimal thresholds for differentiation.

All analyses were performed in the R/Bioconductor environment [16] making use of workflows created in the Duke instance of GenePattern (Broad Institute, MIT, Cambridge, MA).

### Overview of the statistical analysis plan

**Figure 1:**
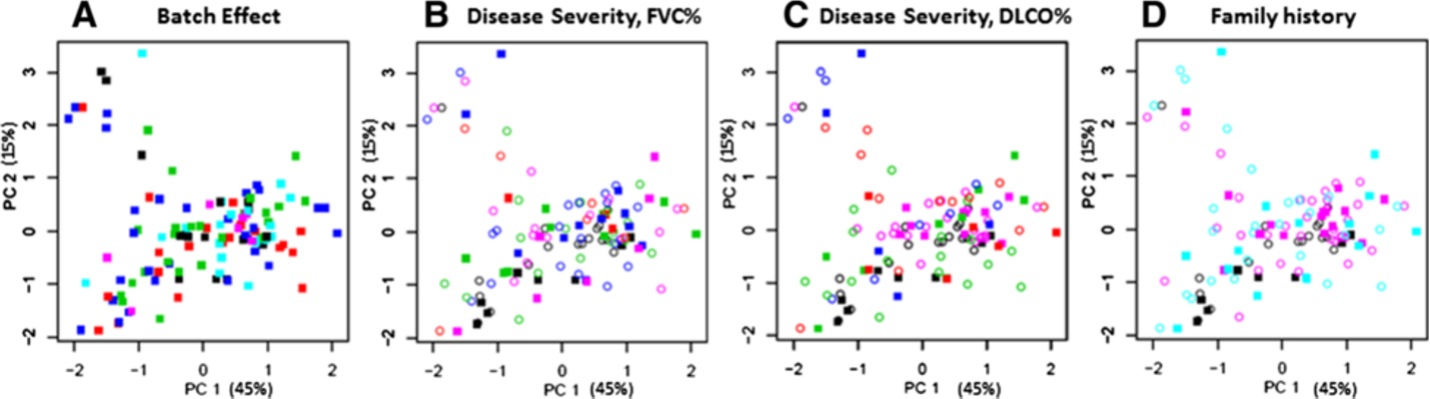
**Principal component analysis of the entire dataset (115 subjects).** First, the data is filtered for genes with a Coefficient of Variation ≥ 90th percentile. Then, all samples are plotted according to expression of the first two Principal Components. **(A)** Samples are identified by batch: batch 1 (*black*), batch 2 (*red*), batch 3 (*green*), batch 4 (*blue*), batch 5 (*cyan*), and batch 6 (*magenta*). **(B)** Samples are identified by severity of disease (FVC%, *see text*): normal (*black*), mild disease (*blue*), moderate disease (*green*), severe disease (*red*), unknown (*magenta*); and the analytic subset: training set (*open circles*), validation set (*closed squares*). **(C)** Samples are identified, again, by the severity of disease (DLCO%): *color code* is the same as in panel **B**. **(D)** Samples are identified by family history: normal (*black*), familial idiopathic pulmonary fibrosis (*cyan*), sporadic idiopathic pulmonary fibrosis (*magenta*).

**Figure 2:**
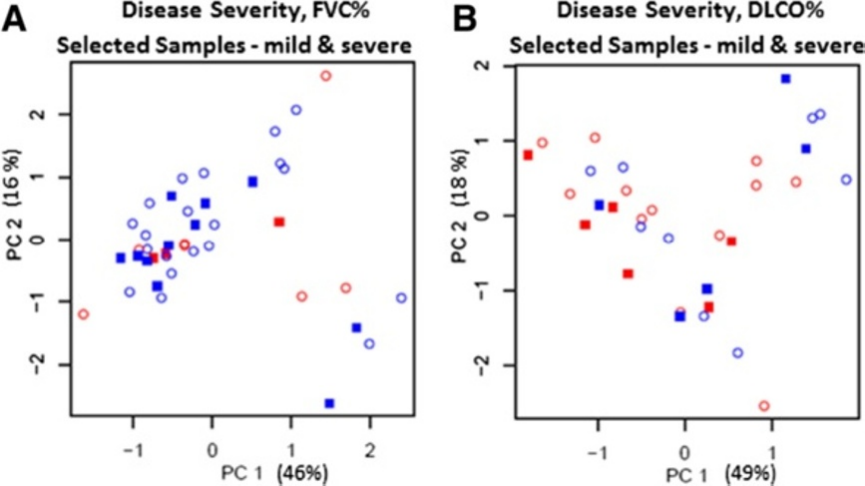
**Principal component analysis of select samples.** Data is filtered by the Coefficient of Variation. Samples are plotted by expression of the first two Principal Components. Key: mild disease (*blue*); severe disease (*red*); training set (*open circles*), validation set (*closed squares*). **(A)** Severity of disease is assessed by FVC% (*see text*): identified 31 cases with mild disease and 10 cases with severe disease. **(B)** Severity of disease is assessed by DLCO%: 14 cases of mild disease versus 18 cases of severe disease.

Prior to developing a gene signature model, the dataset was explored as a whole – to see if there were any global differences in gene expression that might be attributed to batch effects, differences in clinical severity, or family history. This exploratory analysis was performed with an unsupervised method, PCA (Figures 1 and 2). Prior to PCA, the dataset was filtered in an unsupervised fashion using the coefficient of variation (CoV). Filtering was done to improve the signal-to-noise ratio and resulted in a filtered dataset containing only the top 90^th^ percentile by CoV (2208 genes).

The training dataset (two-thirds of the samples) and the validation dataset (remaining one-third) were hierarchically clustered as a means to visually inspect the data for outliers, prior to developing and validating the gene signature model. A 90^th^ percentile CoV filter was applied to both the training dataset and validation dataset prior to hierarchical clustering; again, a filter was applied to improve the signal-to-noise ratio.

Finally, a gene signature model was developed from the training dataset by building consecutive models, over a range of 50–250 genes. Each model was built by using Bayesian probit regression (BPR). Each model was tested for internal validity by using LOOCV. The model with 108 genes had the best internal performance characteristics, measured by area-under-the-curve for misclassification errors, and sum of deviance.

The regression equation from the 108-gene *functional gene signature* model was used to query each sample in the validation dataset. Posterior probability of IPF was determined for each sample in the validation set. Posterior probability of IPF versus the true diagnosis was used to construct a contingency table and evaluate the performance characteristics of the *functional gene signature* as a diagnostic test.

## Results

### Study subjects

Of the 115 samples in this cohort, 48 were obtained from subjects with sporadic IPF; 41 samples were obtained from cases of familial IPF; and 26 samples were obtained from non-diseased healthy controls (Table 1). Forced vital capacity (FVC) and diffusing capacity for carbon monoxide (DLCO) were used to stratify the cases into mild, moderate and severe disease categories. In each stratum, two-thirds of the samples were selected for the training cohort; one-third was reserved for independent validation.

**Table 1:** **Demographics of the study population**

							Disease severity			
	N	Control (%)	Spoaradic (%)	Familial (%)	Age-yrs. (mean ± sd)	Gender (%male)	Mild	Moderate	Severe	Unknown
**Total**	115	26 (23%)	48 (42%)	41 (36%)	66.2 ± 12.1	62	23 (26%)	44 (49%)	14 (16%)	8 (9%)
**Training**	76	17 (22%)	33 (43%)	26 (34%)	66.3 ± 13.4	58	15 (26%)	29 (49%)	9 (15%)	6 (10%)
**Validate**	39	9 (23%)	15 (38%)	15 (38%)	66.1 ± 9.4	69	8 (27%)	15 (50%)	5 (17%)	2 (7%)

### Global analysis of gene expression

#### All samples

The complete dataset (gene expression profile with 22078 Agilent features, Additional file 1) was analyzed to explore relationships among the samples. The dataset was first filtered by ranking genes based on their coefficient of variation. This was done to remove background noise and to enrich the dataset for the most informative genes with the most variation. The top 90th percentile (by coefficient of variation) was retained, resulting in a dataset with 2208 genes.

Then, the filtered dataset was transformed by PCA and the samples were plotted according to expression of the first two principal components. The first two principal components captured 60% of the variation within the filtered dataset (45% is captured by the 1^st^ PC; 15% is captured by the 2^nd^ PC). PCA plots were visually inspected to ascertain the relationship between samples, based on microarray batch, severity of disease and family history (Figure 1).

PCA plots of the complete (filtered) dataset could not distinguish between samples from different batches (Figure 1A). Nor could PCA plots distinguish between samples of different disease severity (Figures 1B and C). (Since there is no consensus as to which pulmonary function parameter is the best, one plot examines gene expression differences across severity defined by FVC while the other plot examines gene expression differences across differences in DLCO). Nor could PCA plots distinguish between samples with different family histories (Figure 1D).

PCA plots were also used to confirm that samples from the training set and validation were essentially indistinguishable (results not shown).

#### Selected samples

The mild cases and the severe cases were picked out and analyzed separately (to reduce background noise) to determine if there were global differences in their gene expression. With severity of disease defined by FVC, there were 31 cases that fit the definition of mild disease and 10 cases that fit the definition of severe disease. PCA plots could not distinguish between the mild and severe cases, as defined by FVC (Figure 2A). With severity of disease defined by DLCO, there were 14 cases that fit the definition of mild disease and 18 cases that fit the definition of severe disease. PCA plots could not distinguish between the mild and severe cases, as defined by DLCO (Figure 2B). Since PCA could not distinguish between sample batches, disease severity defined by either FVC or DLCO, or familial or sporadic cases, the filtered dataset was further analyzed in aggregate.

### Hierarchical clustering and heatmap of the training set

**Figure 3:**
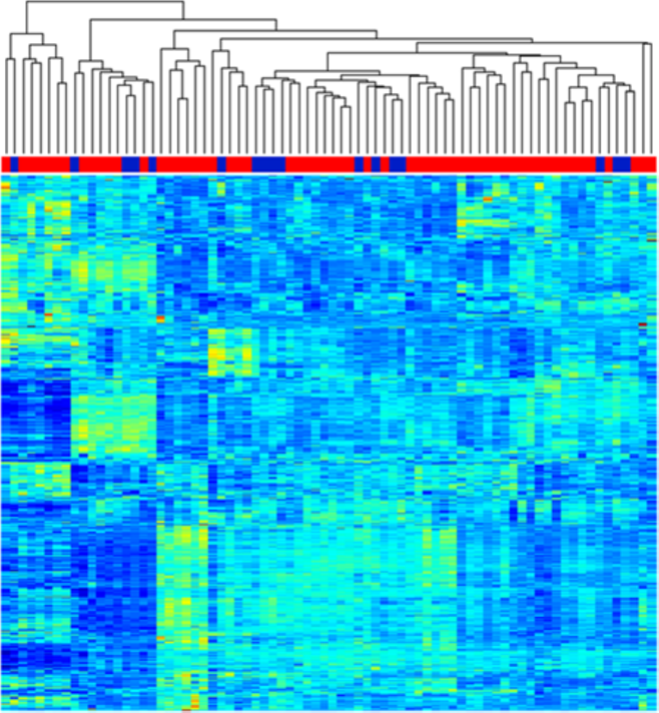
**The** ***training set*** **.** An unsupervised hierarchical cluster of peripheral blood gene expression from 17 normal subjects (*blue*) and 59 IPF subjects (*red*).

The training dataset was examined with hierarchical clustering. The training dataset consisted of 59 samples obtained from the peripheral blood of IPF subjects (including both sporadic and familial cases) and 17 samples obtained from the peripheral blood of normal subjects (Additional file 2). The training dataset was filtered for the top 90th percentile of coefficient of variation (resulting in a dataset with 2208 genes). Visual inspection of the hierarchical cluster could not distinguish between IPF patients and normal subjects (Figure 3).

### Iterative model building

#### Model tuning by leave-one-out cross validation

**Figure 4:**
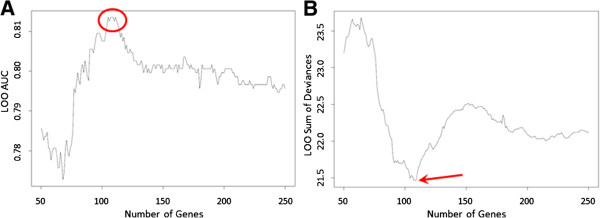
**Selection of features for the model.** Leave-one-out cross validation (LOOCV) is performed on all possible gene signatures, ranging from 50–250 features. Then, the performance characteristics of this bootstrap test are used to select an optimal number of genes (features) with which to build the signature. **(A)** Maximum *area under the curve* is achieved with signatures containing 105, 107, 108, 109 and 111 features. **(B)** The *sum of deviance* of the predicted probabilities is minimized by selecting the signature that contains 108 features.

In developing a Bayesian Probit Regression model for IPF versus normal, one of the first steps is to select the optimal number of features (genes) to include in the functional gene signature. This was accomplished through an iterative data-driven process, whereby consecutive models were constructed, through a range of features from 50–250 genes (the practical limits of computational power). For each consecutive model, internal validity was measured with leave-one-out cross validation (LOOCV) and two parameters were examined: (a) the rate of phenotype misclassifications, calculated by measuring the area under the receiver operating characteristic curve (ROC statistic); and (b) the sum of deviance (SOD), an aggregate of deviances between the predicted posterior probability and the expected posterior probability of the true phenotype for each sample. The ROC statistic identified five potential models (with maximal performance on the LOOVC test): functional gene signatures containing 105, 107, 108, 109 and 111 features all attained ROC statistic = 0.814. Among these potential signatures, the optimal functional gene signature was chosen by examining the SOD. The functional gene signature with 108 gene features had the least SOD = 21.461; signatures containing 107 and 109 genes were close, with SOD = 21.469 and SOD = 21.467 respectively. The 108 gene signature was considered most valid, by a combination of ROC and SOD criteria (Figure 4).

### The diagnostic gene signature

It was determined that 108 genes provide the optimal number of features for a functional gene signature derived from this particular training set, using the internal validation procedures described above.

**Figure 5:**
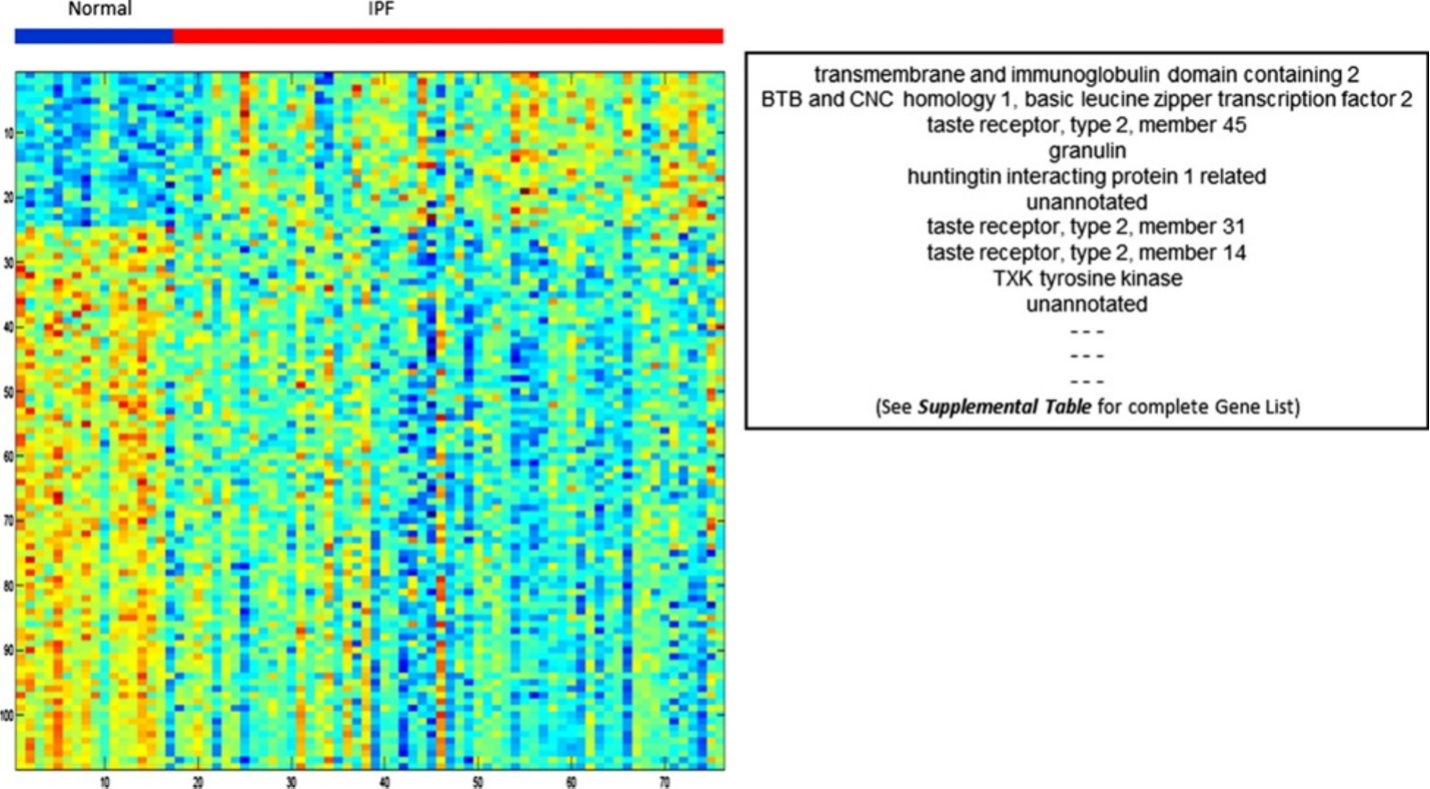
**The peripheral blood gene signature.** A heatmap displays the normalized expression values of 108 genes that comprise the model, derived from the peripheral blood of 17 normal controls and 59 subjects with IPF. A partial gene list (top ten genes) is displayed alongside the heatmap. The complete gene list (all 108 genes) is provided in the online supplement.

The gene signature is visualized with a heatmap that highlights the necessary components of any functional gene signature: training samples (across the columns), gene features (across the rows) and the actual expression values (upon which the model is constructed) that are unique to this dataset (Figure 5). The complete regression equation (with intercept and gene coefficients) is provided in the online supplement (Additional file 3). This regression equation can be used to predict the phenotype in any unknown sample. Thus, the diagnostic gene signature presented herein can be used to inform the diagnosis of IPF versus normal in any patient, using the gene expression profile derived from peripheral blood.

A partial list of the genes (features) that comprise this gene signature is shown in Figure 5. The complete list of 108 features is included in the online supplement (Additional file 4).

### Independent validation

#### Validation dataset

**Figure 6:**
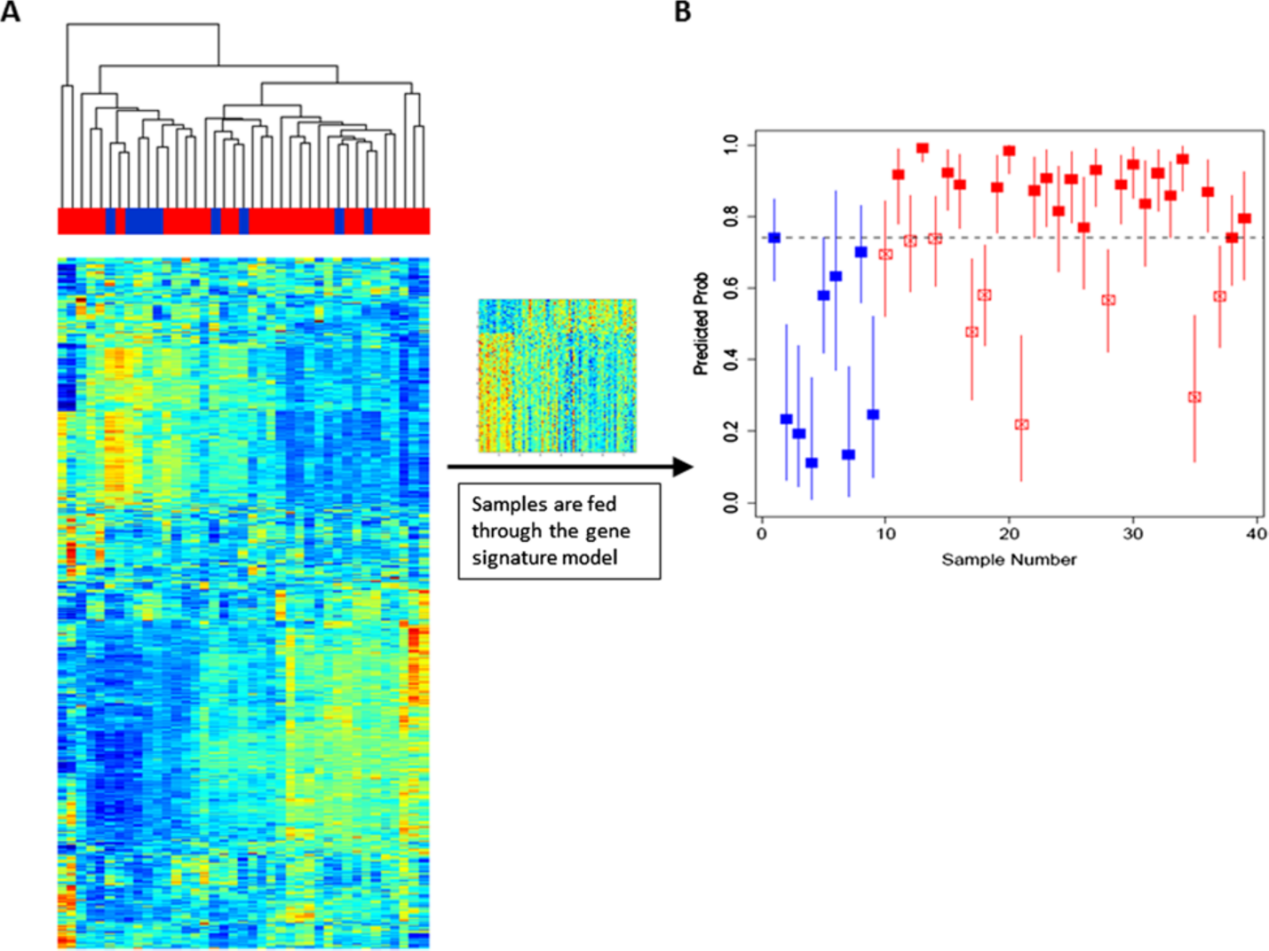
**Validation testing. (A)** Unsupervised hierarchical clustering of the *validation set*, includes 9 peripheral blood samples from normal subjects (*black*) and 30 samples from IPF subjects (*red*). **(B)** Each sample from the *validation set* is assigned a probability of IPF, and a credible interval to that value (solid bars), by Bayesian modeling to the gene signature. Normal subjects (*blue*) tend to receive low probability scores while IPF subjects (*red*) tend to receive high probability scores. The optimal threshold for this test was determined by the Youden index (*dotted line*).

Figure 6A shows hierarchical clustering of the validation dataset. The validation dataset includes 30 samples from IPF subjects (familial and sporadic) and 9 samples from normal subjects (Additional file 5). The validation dataset was filtered by coefficient of variation. Visual inspection shows that hierarchical clustering of the complete (filtered) validation dataset does not distinguish IPF from normal.

#### Validation test

Each sample in the validation set was fed into the gene signature model which produces a *predicted probability* (the likelihood of an IPF diagnosis for this particular sample). Predicted probabilities of all samples are shown in Figure 6B. Predicted probabilities are compared against the true phenotype. In general, normal samples receive a low or moderate *predicted probability*; while true IPF samples are assigned high *predicted probabilities*.

**Figure 7:**
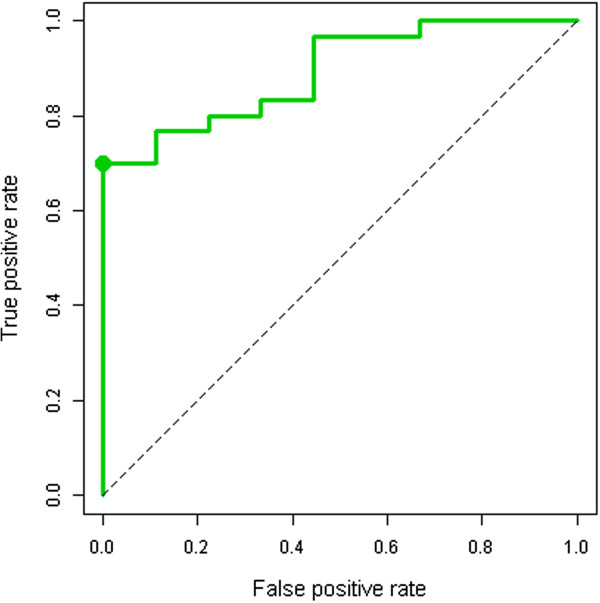
**ROC curve, based on results of the validation testing.** The optimal cutoff point (as indicated) was determined by the Youden index. See Table 2 for a numerical summary of the operating characteristics.

An ROC curve (Figure 7) and a contingency table (Additional file 6) are used to analyze the performance characteristics of this gene signature, based on results of the validation test. The Youden index was used to compute the optimal threshold of *predictive probability* that maximizes sensitivity and specificity. The Wilcoxon rank sum was performed to test the general association between *predicted probability* and true phenotype (p < 0.0001). Complete performance characteristics (Table 2) show a sensitivity of 70%, specificity of 100%, positive predictive value of 100%, negative predictive value of 50%, and overall accuracy of 77%.

**Table 2:** **Operating characteristics of the peripheral blood gene signature**

Area under the curve	Optimal cutoff	Sensitivity	Specificity	Positive predictive value	Negative predictive value	Overall accuracy	Wilcoxon rank-sum (p-value)
0.893	0.74	70%	100%	100%	50%	77%	< 0.0001

## Discussion

We compared peripheral blood transcriptomes from patients with IPF and normal controls; and we derived and validated a blood-based *functional gene signature* that distinguishes IPF from normal. In a tertiary care population such as the one included in our study, our gene signature is 77% accurate; with sensitivity estimated at 70% and specificity estimated at 100% (area under the ROC curve is 0.893). Wilcoxon rank sum demonstrates the general discriminative ability of our gene signature (p < 0.0001).

Previously, we showed how BPR-methods could be used to derive a *functional gene signature* from whole lung tissue. Now, we present a validated, diagnostic IPF gene signature derived from the peripheral blood transcriptome. This tool has the potential to improve diagnostic accuracy while offering a less invasive approach to the diagnosis of IPF.

In the course of these experiments, we also explored the relationship between gene expression profiling and disease severity (as measured by FVC or DLCO). We found that global expression profiles could not sufficiently discriminate between mild, moderate, and severe disease. Therefore, we decided to ignore these factors in developing our predictive model.

This report is a secondary analysis of data previously reported by Yang *et al.*[13]. While the analysis performed by Yang *et al.* was focused on describing differentially expressed genes in this cohort, our goal was quite different. We aimed to develop and validate a predictive classification model for the diagnosis of IPF. Interestingly, both our analysis and the Yang analysis suggest that granulin (GRN) and matrix metalloprotease 9 (MMP-9) play an important role in the biology of IPF. GRN and MMP-9 expression are both increased in IPF as compared to normal controls. MMP-9 is a well-known gene; its protein product has already been implicated in the pathogenesis of IPF. [17] GRN has never before been described in IPF; its product, progranulin, is a growth factor that plays important roles in cancer biology, tissue remodeling, neurodegenerative disease and hepatic fibrosis [18, 19].

Excessive fibroproliferation in the lung leading to excessive collagen deposition is characteristic of IPF, and several of the genes in our 108 gene signature may play a role in this process. 1-Acylglycerol-3-phospate O-acyltransferas 9 (AGPAT9) is a member of the lysophosphatidic acid aceyltransferase protein family, an enzyme which catalyzes the conversion of glycerol-3-phosphage into lysophosphatidic acid (LPA) in the synthesis of triacylglycerol. LPA is increased in bronchoalveolar lavage fluid following lung injury in the bleomycin model of pulmonary fibrosis, and mice lacking of its receptors, LPA1, are protected from injury [20]. Currently, a LPA1 receptor antagonist is in clinical trial as a potential treatment for IPF. In this study, AGPAT9 is up-regulated in the blood of patients with IPF, and it is possible that circulating mononuclear cells maybe an important source of LPA promoting lung fibrosis. There are several lines of evidence that indicate alveolar epithelial integrity and dysfunction are important in the pathobiology of IPF. [21] There is particular interest in the role of chronic herpesvirus infection may play in promoting alveolar epithelial cell dysfunction in IPF [22], and it is of interest that CD79B, a component of the B lymphocyte antigen receptor, is up-regulated gene in our signature. Peripheral blood cells migrating through the lung releasing profibrotic proteins such as MMP9, granulin, or AGPAT9, or lymphocytes interacting with herpesvirus in the lung, shows how a blood-based RNA signature could reflect disease processes in the lung. It is also important to remember that 98-99% of the cardiac output circulates through the lung whereas most organs only receive much smaller fraction, therefore processes in the circulation may have a greater impact on the lung. Similarly, cross-talk between circulating cells and the lung may be greater than in other organs.

We acknowledge several limitations of this study. For instance, our gene signature has so far only been validated on independent samples. Though independent, these samples came from the same population as the training samples. Our gene signature is yet to be validated on an external population. Thus, performance characteristics in the general population remain unknown. It will also be important to test the gene signature on a greater number and wider variety of patient samples to determine if confounders such as presence of infection, disease severity or disease stability influence performance of the signature.

Nevertheless, we have demonstrated the possibility of diagnosing IPF from the peripheral blood. We plan to validate our current gene signature in large external populations to determine if the signature retains its predictive capacity in heterogeneous IPF populations. This test may be of great value in identifying and diagnosing early disease in our familial pulmonary fibrosis cohort that contains over 800 families collected across the U.S., many with incomplete phenotype information. We also propose the feasibility to develop other blood-based gene signatures that may improve diagnostic accuracy across the entire spectrum of ILD from nonspecific interstitial pneumonia to hypersensitivity pneumonitis and so forth. In addition, blood-based gene signatures could be used in conjunction with information about IPF gene risk variants [23].

### Ethics

All research involving human subjects, human material, and human data has been performed in accordance with the Declaration of Helsinki, and with approval of ethics committees at Duke University Medical Center, National Jewish Health, and University of Colorado Denver Health Sciences Center.

## Conclusions

We derived and validated a blood-based *functional gene signature* that distinguishes IPF from normal. In a tertiary care population such as the one included in our study, our gene signature is 77% accurate; with sensitivity estimated at 70% and specificity estimated at 100% (area under the ROC curve is 0.893). Wilcoxon rank sum demonstrates the general discriminative ability of our gene signature (p < 0.0001).

## Electronic supplementary material

Additional file 1: **The complete dataset, pre-processed.** (XLS 43 MB)

Additional file 2: **The training set, subset of the complete dataset.** (XLS 29 MB)

Additional file 3: **The validation set, subset of the complete dataset.** (XLS 15 MB)

Additional file 4: **Intercept and coefficients of the regression equation.** (XLS 23 KB)

Additional file 5: **The gene list.** This file includes gene names; average expression values across all the IPF samples and normals; log-fold change in expression value between the IPF samples and normals; and the FDR-adjusted p-values for Student’s t-test comparing the IPF samples to the normal controls. (XLS 46 KB)

Additional file 6: **Contingency table.** (PPTX 73 KB)

## Abbreviations

IPF: Idiopathic pulmonary fibrosis
FIP: Familial interstitial pneumonia
BPR: Bayesian probit regression
FVC: Forced vital capacity
DLCO: Diffusing capacity for carbon monoxide
MCMC: Monte Carlo Markov Chain
ROC: Receiver operator characteristic
LOOCV: Leave-one-out cross validation
AUC: Area under curve
SOD: Sum of deviance.
