# Supplementary material for: Familial and sporadic idiopathic pulmonary fibrosis: making the diagnosis from peripheral blood
Source: BMC Genomics. 2014 Oct 16;15(1):902. doi: 10.1186/1471-2164-15-902 (PMC4288625; doi:10.1186/1471-2164-15-902)
Supplement: Supplementary file 6 — Additional file 6: Contingency table. (PPTX 73 KB) [file 12864_2014_6786_MOESM6_ESM.pptx]

## Slide 1
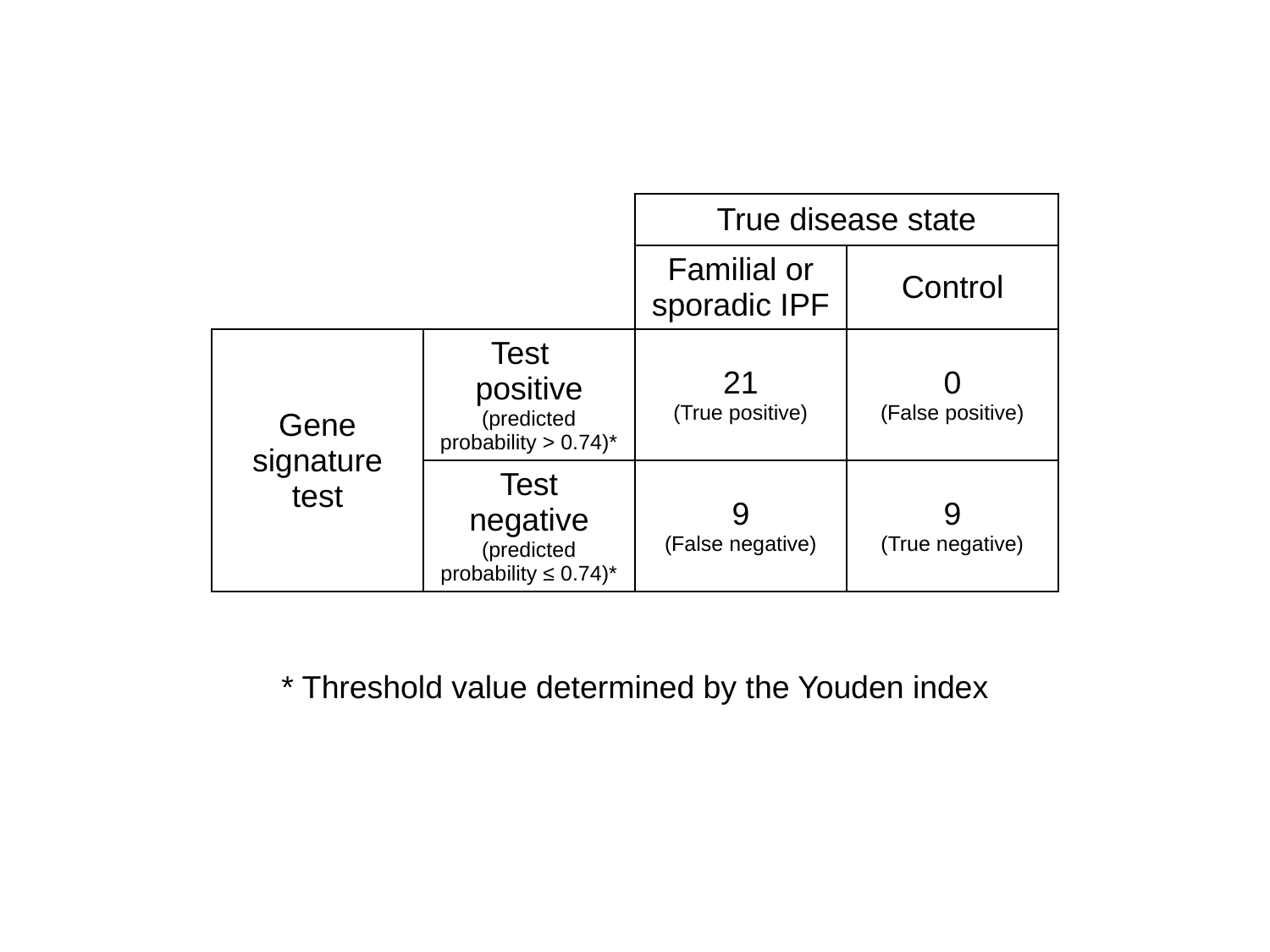

| | | True disease state | |
| --- | --- | --- | --- |
| | | Familial or sporadic IPF | Control |
| Gene signature test | Test positive (predicted probability > 0.74)\* | 21 (True positive) | 0 (False positive) |
| | Test negative (predicted probability ≤ 0.74)\* | 9 (False negative) | 9 (True negative) |
* Threshold value determined by the Youden index
